# Automated Protein Biomarker Analysis: on-line extraction of clinical samples by Molecularly Imprinted Polymers

**DOI:** 10.1038/srep44298

**Published:** 2017-03-17

**Authors:** Cecilia Rossetti, Magdalena A. Świtnicka-Plak, Trine Grønhaug Halvorsen, Peter A.G. Cormack, Börje Sellergren, Léon Reubsaet

**Affiliations:** 1 Department of Pharmaceutical Chemistry, University of Oslo, School of Pharmacy, Postbox 1068 Blindern, 0316 Oslo, Norway; 2 WestCHEM, Department of Pure and Applied Chemistry, University of Strathclyde, Thomas Graham Building, 295 Cathedral Street, Glasgow, G1 1XL, United Kingdom; 3 Department of Biomedical Sciences, Faculty of Health and Society, Malmö University, SE20506 Malmö, Sweden

## Abstract

Robust biomarker quantification is essential for the accurate diagnosis of diseases and is of great value in cancer management. In this paper, an innovative diagnostic platform is presented which provides automated molecularly imprinted solid-phase extraction (MISPE) followed by liquid chromatography-mass spectrometry (LC-MS) for biomarker determination using ProGastrin Releasing Peptide (ProGRP), a highly sensitive biomarker for Small Cell Lung Cancer, as a model. Molecularly imprinted polymer microspheres were synthesized by precipitation polymerization and analytical optimization of the most promising material led to the development of an automated quantification method for ProGRP. The method enabled analysis of patient serum samples with elevated ProGRP levels. Particularly low sample volumes were permitted using the automated extraction within a method which was time-efficient, thereby demonstrating the potential of such a strategy in a clinical setting.

Automated biomarker analysis is attracting significant attention in the field of proteomics^1^. Furthermore, biomarker analyses which require low sample volumes and minimal sample handling steps are of particular interest in clinics. Very often, it is the limited availability of sample together with the need for a reliable, cost- and time-effective method which leads to “conventional” immunoassays being preferred over innovative mass spectrometry (MS) assays^2^. In recent years, there has been an intense focus upon automated systems directly integrating sample preparation with MS bioanalysis to satisfy clinical requirements^3^,^4^,^5^. Within this context, many efforts have been made to develop reliable and sensitive MS alternatives to immunoassays for biomarker quantification, including MS assays for the low abundant biomarker ProGastrin Releasing Peptide (ProGRP) which has been studied widely as model biomarker^6^,^7^,^8^,^9^,^10^,^11^,^12^.

ProGRP is a sensitive (reference level of 7.6 pM in serum) and specific biomarker with diagnostic and prognostic value for Small Cell Lung Cancer (SCLC)^13^,^14^,^15^,^16^,^17^,^18^. Hence, quantitative information on its abundance in serum will strongly impact SCLC management.

Currently, ProGRP is analysed in the clinics by ELISA with a time-resolved immunofluorometric assay (TR-IFMA)^19^. However, targeted proteomic immuno-MS assays^10^,^11^ have also been developed, allowing the quantification of ProGRP through a bottom-up approach. The use of immunoextraction prior to the MS analysis was shown to be essential for the realization of low detection limits, to enable discrimination between healthy and patient donors according to ProGRP expression. Other studies have focused on ProGRP extraction with the aim of replacing antibodies with synthetic receptors^20^,^21^. In this regard, molecularly imprinted polymers (MIPs) were developed and used as “plastic antibodies” for the off-line enrichment of the ProGRP signature peptide (NLLGLIEAK) from serum. This method was well-suited for coupling with the MS assays developed previously^11^ and represented a fast and economical alternative to immunocapture. However, the off-line MIP extraction was unable to determine ProGRP concentrations close to the reference level due to the high detection limits of the off-line method^21^. The MIPs used in the aforementioned study were synthesized *via* a template analogue imprinting strategy, a powerful approach reported previously by Manesiotis *et al*.^22^. In the case of ProGRP, an analogue of the signature peptide was used as template in the production of thin MIP films on silica beads surfaces *via* a non-covalent molecular imprinting protocol, giving core-shell RAFT-beaded particles.

The use of uniform, beaded MIPs is particularly desirable in challenging separation science applications since beads are physically robust and can be easily and reproducibly packed into solid-phase extraction (SPE) cartridges and chromatographic columns, circumventing any fluid flow problems arising from high back pressures^23^,^24^,^25^. Uniform, beaded MIPs with low mean particle sizes offer yet further advantages since the low particle size leads to high separation efficiencies thanks to the fast binding kinetics arising from improved accessibility of binding sites^26^.

Precipitation polymerization is a very attractive synthetic method for the synthesis of MIP microspheres^27^,^28^,^29^,^30^,^31^,^32^,^33^. MIP microspheres of controlled size and porosity are obtained easily by the tuning of polymerization conditions^34^,^35^,^36^ without the need for surfactants or stabilizers, delivering clean products with narrow particle size distributions. Typically, the microspheres are obtained in one synthetic step and the particle diameters are normally in the range 0.1–10 μm^37^. MIP microspheres are thus particularly well-suited as molecularly selective packings in trap columns for integration with MS systems, as will be demonstrated in this study.

Within this context, molecularly selective polymeric sorbents were prepared by precipitation polymerization to develop an innovative diagnostic approach for ProGRP quantification, involving automated MIP-based extraction coupled with liquid chromatography-MS (LC-MS). The main goal was to evaluate the performance of the new, automated MIP extraction method on patient serum samples containing clinically relevant concentrations of ProGRP.

## Results and Discussion

### Synthesis of MIPs: template, functional monomers and crosslinker selection

The template used was Z-NLLGLIEA[Nle]; in effect, the N-terminus of the signature peptide has been protected with a benzyloxycarbonyl group (Cbz; Z) to enhance the solubility of the template in the porogenic solvents used for the polymerization, and the C-terminal lysine has been replaced by norleucine (Nle)^21^. The latter modification was introduced in order to overcome the intramolecular competition for the anionic sites caused by the lysine side.

**Figure 1:**
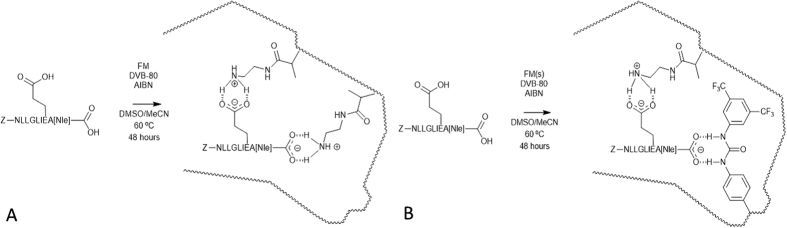
Representation of the non-covalent molecular imprinting of Z-NLLGLIEA[Nle] for MIP A (**A**) and MIP B (**B**). The carboxylic acid groups in the glutamic acid (E) residue and C-terminus of Z-NLLGLIEA[Nle] are drawn explicitly for emphasis, since these functional groups are involved in the self-assembly of the Z-NLLGLIEA[Nle] with the functional monomers (FMs). The complexed synthetic receptors depict the hypothetical molecularly imprinted binding sites formed upon the free radical copolymerisation of a molecular complex of Z-NLLGLIEA[Nle] and FM(s) with crosslinker (DVB-80).

Two different functional monomers were used, N-(2-aminoethyl)methacrylamide hydrochloride (EAMA.HCl), solely for MIP A, and *N*-3,5-*bis*(trifluoromethyl)-phenyl-*N*′-4-vinylphenylurea,together with EAMA.HCl for MIP B, since the carboxylic acid groups in the glutamic acid (E) residue and C-terminus of the template were targeted *via* a non-covalent molecular imprinting approach. Indeed, both monomers have been shown to be useful for the targeting of oxy-anions^20^,^38^,^39^. A representation of the Z-NLLGLIEA[Nle]-imprinted binding sites in MIP A and MIP B is shown in Fig. 1A and B, respectively. For success, precipitation polymerizations must involve the polymerization of monomers in dilute solution (typically <5% w/v monomer in solvent) in a near-⊖ solvent, therefore DVB-80 was selected as crosslinker, the porogen was MeCN and the monomer concentration was fixed at 2% w/v. DMSO was required to promote solubility of the template, but the use of this dipolar aprotic solvent was kept to a minimum (Supplementary Table S-1).

High crosslinker levels were used to ensure good yields of mechanically robust polymer microspheres with well-developed and permanent porous morphology. The mole ratio of template to FMs was set at 1:10 (Supplementary Table S-3). This small excess promotes template-FM self-assembly, minimizing the possibility of non-specific binding events arising from the random incorporation of a large excess of FMs into the polymer networks, as reported previously^39^. Moreover, the choice of precipitation polymerization as synthetic protocol yielded uniform, porous, particles with low mean particle diameters (as shown below) suitable for packing into the trap columns, without any need for the silica-core which was the inner component of the larger RAFT-MIPs for ProGRP (20 μm silica-core particles) reported earlier^20^,^21^.

### Characterization of the polymers

The SEM micrographs of the polymers (Supplementary Fig. S-2) revealed the production of discrete particles in the low micron-sized range (diameters ≤5 μm), although the microspheres were polydisperse (possibly as a consequence of the presence of DMSO as a co-solvent). The nitrogen sorption data (Supplementary Table S-4) revealed that the MIPs and NIPs were porous, but mean pore diameters placing them at the boundary between microporous and mesoporous solids; this was important to establish in view of the need for analyte to access molecularly imprinted binding sites during the SPE. The NIPs were not identical to the MIPs in respect of their porous morphologies, indicating an influence of the template on the timing of the phase separation^40^. Although this is often the case for MIP/NIP pairs, since by definition a NIP is synthesised in the absence of any template whatsoever and there can be no template influence upon the polymerization, the differences are probably accentuated here because we are operating close to the solubility limit of the template. Irrespective of the morphology differences, however, through careful optimisation binding conditions which enabled binding affinity and selectivity could be established.

### Peptide retention on MIP and corresponding NIP by direct injection of ProGRP isoform 1

**Figure 2:**
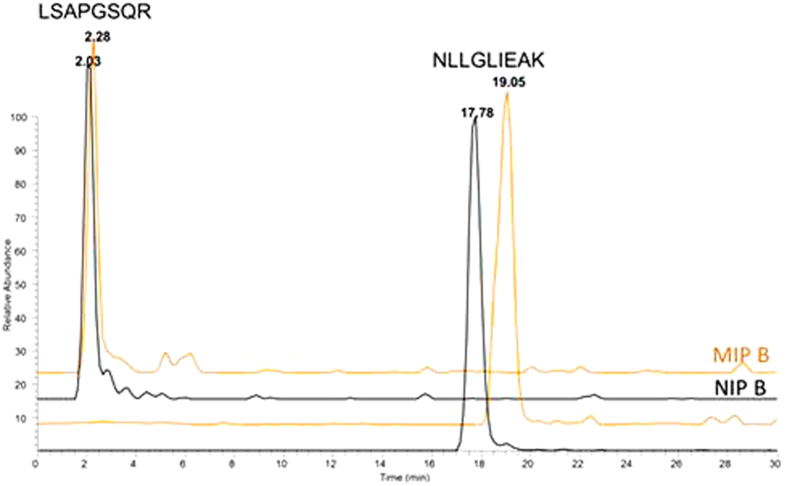
MS/MS Chromatograms of 10 nM digested ProGRP isoform 1 obtained by using MIP B (orange) and NIP B (black) coupled directly to the MS detector without analytical column.

All four polymers were packed into stainless steel columns and evaluated for peptide retention by direct injection of protein digests containing the target peptide NLLGLIEAK. Thus, ProGRP isoform 1 was trypsinated and loaded on the MIP and NIP columns which were, at this stage, used as analytical columns coupled directly with the ESI source of the MS detector (Fig. 2). The SRM transitions corresponding to the ProGRP peptides (LSAPGSQR and the target peptide NLLGLIEAK) were acquired from the moment of the injection to the end of the gradient. No retention was seen for the signature peptide of isoform 1 of ProGRP (LSAPGSQR) on both MIP and NIP columns. The target peptide, NLLGLIEAK, was retained longer on the MIP (19.05 minutes), and this was reassuring given the intention to use the MIP as a trap column in a later part of the study.

The corresponding NIP B also showed affinity for NLLGLIEAK and this can be ascribed to non-covalent interactions between this peptide and the polymer. Similar retention differences and trends were observed when MIP A and NIP A were tested.

### Effect of the loading pH

**Figure 3:**
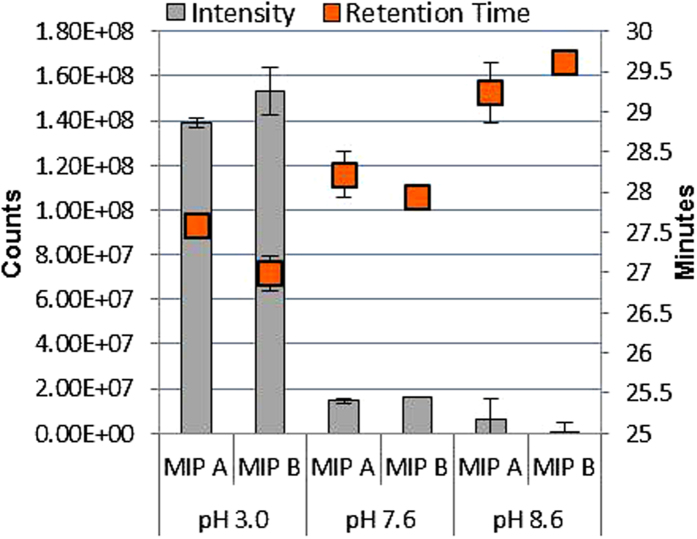
Effect of loading pH on retention times and peak areas of NLLGLIEA[K_^13^C_6_^15^N_2_] (5 nM) extracted on both MIPs.

The optimal pH to promote non-covalent interactions between the target peptide and the binding sites of the MIPs was assessed by testing MIP A and B solely. Figure 3 shows the retention time and the intensities obtained on both MIPs upon loading the heavy labelled target peptide NLLGLIEA[K_^13^C_6_^15^N_2_] at three different pH values (3.0, 7.6 and 8.6). Loading with 20 mM FA (pH 3.0) for 10 minutes gave peptide high intensity and retention times above 27 minutes on both MIPs. Upon increasing the pH of the loading solution using 50 mM ammonium bicarbonate buffer adjusted to pH 7.6 and pH 8.6, the retention time of the peptide rises until 29.5 minutes, but a drop in signal intensity is observed simultaneously for both MIPs. The increase in peptide retention at higher pH can be rationalized as the progressive strengthening of the interactions between the positively charged EAMA residues in both polymers (p*Ka* 9.6) and the negatively charges of the glutamic acid residue (p*Ka* 4.2) and the C-terminal carboxylic acid (p*Ka* 2.2) of the peptide. At pH 3.0 only 10% of the glutamic acid residues are charged while for pH >6.2 more than 99% of them are available to establish ionic interactions with the FM^41^. Likewise, 90% of the C-terminal carboxylic acid is charged at pH 3.0 contributing to the peptide retention which increases at higher pH.

In addition to these interactions, a combined effect of the peptide negative charges (pI 6.44) is feasible when the pH is basic. The drop in signal intensities can be ascribed to incomplete positive ionization of the peptide in the MS detector when the pH is >7. This was confirmed by direct injection in the TSQ analyzer of the peptide solutions (1 nM) with three different pH values (3.0, 7.6 and 8.6) (Supplementary Fig. S-5). Since the increase in retention time at higher pH was of less significance than the increase in signal intensity at low pH, 20 mM FA was used for the loading of the samples on the columns.

### Evaluation of MIP/NIP pairs and MIP selection

**Figure 4:**
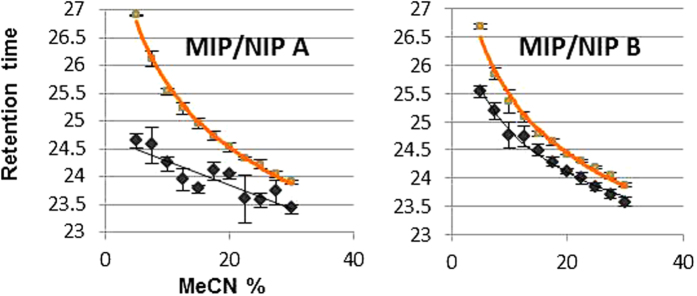
Differences in retention times of NLLGLIEA[K_^13^C_6_^15^N_2_] (1 nM) on the MIPs (orange) and corresponding NIPs (black) for both polymer pairs (A and B).

**Figure 5:**
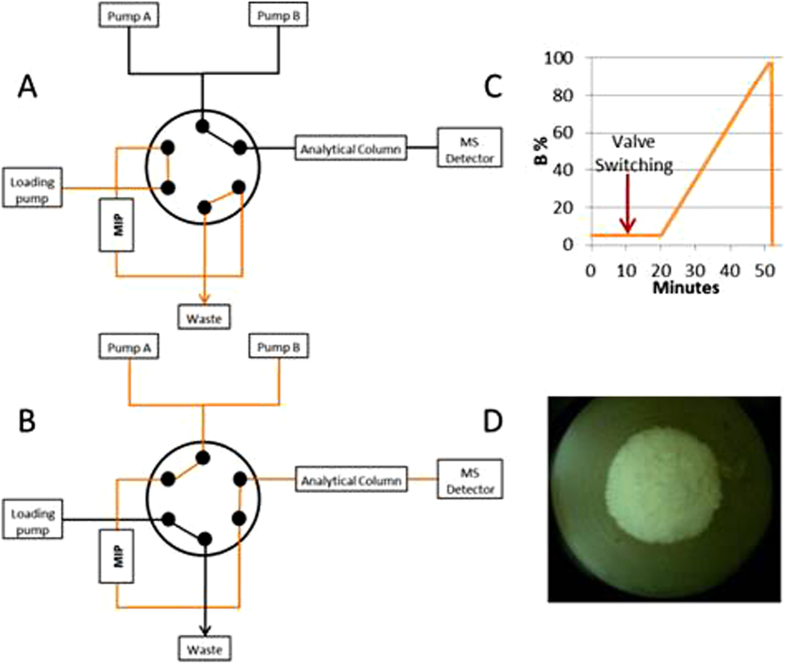
Schematic representation of on-line extraction using a 6-port-valve: (**A**) loading of the sample on MIP column, (**B**) forward-flushing of the MIP column to the analytical column, (**C**) analytical gradient applied for NLLGLIEAK determination, (**D**) transversal section of MIP A after packing in the trap column.

The imprinting effects were evaluated by comparison of the NLLGLIEAK retention times on the two MIP/NIP pairs. Retention times of NLLGLIEA[K_^13^C_6_^15^N_2_] were recorded upon its loading onto all the columns with 10 column volumes of 20 mM FA and subsequent isocratic elution directed to the MS detector, using small MeCN increments (Fig. 4). The differences in NLLGLIEA[K_^13^C_6_^15^N_2_] retention of the MIP/NIP pairs appears to be highest when EAMA.HCl was used as sole functional monomer (MIP A). Any significant differences in peptide retention among the polymers batches can be ascribed uniquely to differences in the structures of the binding sites, since the columns were checked for complete packing by optical control of the transversal section of the cartridges (Fig. 5D) and measurement of backpressures gave similar results for all columns (7 PSI for MIP A and NIP A and 10 PSI for MIP B and NIP B).

The MIP A column was selected as trap column for further automatization and coupled with the analytical column. The MIP A column gave longer analyte retention, which is desirable for highly specific enrichment of the peptide when it is in the presence of many different interferences occurring in complex matrices such as serum samples. Additionally, MIP A showed a higher imprinting factor (IF) than MIP B (Supplementary Table S-6) under the conditions of use. These MIPs are distinct to many others synthesized by precipitation polymerisation, in that the low solubility of the template in the porogen necessitated the use of low template concentrations and high crosslink ratios (Supplementary Table S-3). Such synthetic constraints lead to MIPs with theoretical binding capacities that are considerably lower than MIPs synthesised under traditional conditions, and imprinting factors that are deceptively low when the MIPs are evaluated under normal loading conditions. The modest binding capacity of the MIPs is not a concern given that the concentrations of the target in the clinical samples is in the pM range, since the MIPs will not be over-loaded when in use (under the conditions of use of the MIPs for the clinical samples, a proportion of the highest fidelity binding sites are being exploited) and binding conditions that enabled binding affinity and selectivity could be established.

### Coupling of MIP columns with the analytical column and method optimization

The arrangement of the 6-port valve when the sample is loaded onto the MIP column and subsequent valve switching is shown in Fig. 5A and B respectively.

**Figure 6:**
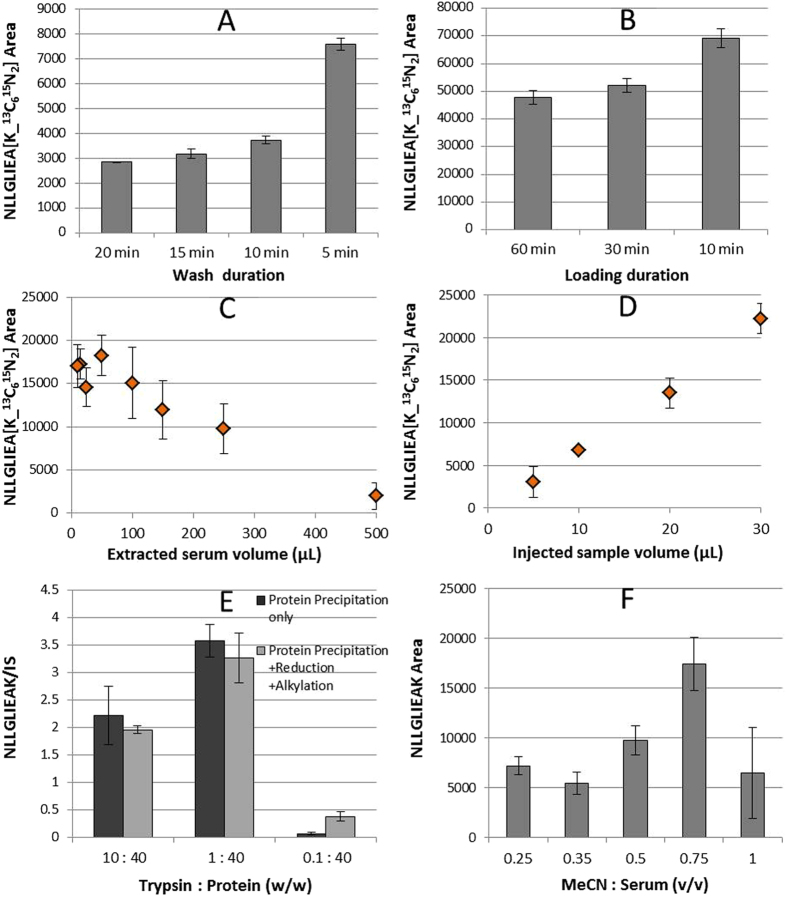
Extraction optimization (**A**–**D**) by using 1 nM NLLGLIEA[K_^13^C_6_^15^N_2_]: (**A**) duration of the wash step (5% MeCN) on MIP A column; (**B**) duration of the loading step (20 mM FA) on MIP A column; (**C**) capacity evaluation by extraction of different serum volumes; (**D**) injection volume evaluation by extraction of 50 μL of serum. Sample pretreatment optimization (**E**-**F**) by using 37 nM ProGRP isoform 1 spiked serum samples: (**E**) evaluation of trypsin amount and reduction (DTT) and alkylation (IAA) after protein precipitation (PP) on spiked serum; (**F**) optimization of the MeCN:serum ratio (v/v) in protein precipitation step.

Optimization of the wash and loading duration (Fig. 6A and B) provided 10 minutes for loading and washing for 5 minutes, whilst keeping the flow constant at 30 μL/min. The capacity of the columns determined the serum volume to be extracted (Fig. 6C). The extraction of 50 μL of serum performed remarkably well in terms of peptide signal intensity (for comparison, the present gold standard method TR-IFMA requires 100 μL) and was judged to be optimal. This result was very promising indeed for the management of clinical samples which are often available in very limited volumes only. Increasing the injection volume from 5 to 30 μL allowed a linear increase in the peptide signal intensity (Fig. 6D), demonstrating the suitability of the extraction of 50 μL of serum. In order to minimize the sample complexity before the extraction, depletion of the high abundant proteins such as serum albumin was decided to be performed by protein precipitation. This step was optimized by testing different MeCN volumes for the protein precipitation of ProGRP isoform 1 spiked samples. The highest peptide recovery was achieved using a 0.75:1 ratio of MeCN:serum (v/v) and 1:40 trypsin to substrate ratio, without reduction/alkylation (Fig. 6E and F). The enzyme to protein ratios shown in the figure are based on the amount of serum albumin expected to be left in the sample after protein precipitation. The amounts ranged between 1 and 10% in earlier studies which investigated protein precipitation with different acetonitrile concentrations^42^,^43^. Accordingly, a depletion of at least 90% of serum albumin with 50% of acetonitrile as precipitant agent can be assumed.

The extraction into the on-line system and the chromatographic run were complete within 50 minutes. The overall outcome was an automated and cost-effective method with remarkably low sample volume consumption.

### Linearity, LOD and LOQ

The linearity of the method was explored over 3 orders of magnitude of ProGRP levels. The regression curve obtained (Supplementary Fig. S-6) upon plotting the ratio of the area of the signature peptide NLLGLIEAK to the area of the IS NLLGLIEA[K_^13^C_6_^15^N_2_] had an acceptable correlation value (R^2^ > 0.97).

From the signal-to-noise ratio (S/N) of the lowest concentration of the curve, the limit of detection (LOD) was estimated to be 17.2 pM (S/N = 3) corresponding to a lower limit of quantification (LLOQ) of 57.3 pM (S/N = 10). The mass limit of detection (mLOD) on column was estimated to be 425 amol.

The detection limit of this new method is therefore substantially lower than the limit achieved previously by the MIP-based extraction^21^ (625 pM) but is still marginally higher than the immunocapture LC-MS^10^ (1 pM) and TR-IFMA methods. In the case of extended disease, clinically relevant concentrations of ProGRP are above the LOD achieved with this method^44^. However, the method is not able to discriminate healthy donors close to the reference limit of 7.6 pM.

### Analysis of patient samples and benchmarking with other methods

**Figure 7:**
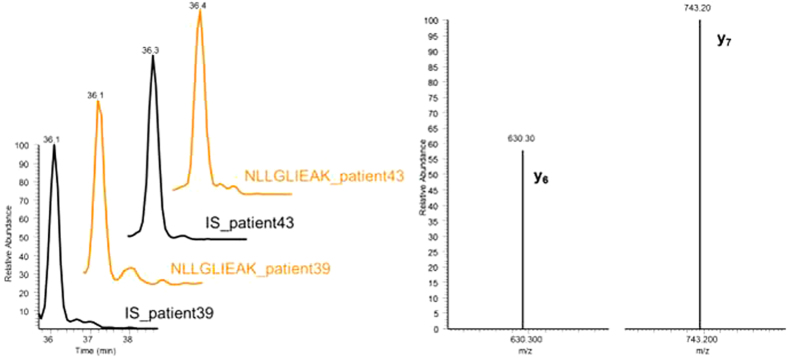
Analysis of patient serum samples: Chromatograms of NLLGLIEAK (orange) and the Internal Standard (IS) NLLGLIEA[K_^13^C_6_^15^N_2_] (black) (left side) and corresponding ion spectra for selected reaction monitored (fragments y_6_ and y_7_) for NLLGLIEAK determination (right side).

Two patient serum samples suffering from SCLC were analyzed to demonstrate the applicability of the method to determine ProGRP in real samples with high levels of endogenous ProGRP (Fig. 7). The monitoring of selected transitions of NLLGLIEAK together with the co-elution of the IS allowed a correct peak identification.

From the calibration curve, the ProGRP concentrations were calculated for both samples; the values are reported in Table 1 together with the ProGRP concentrations determined previously for these samples by the immunocapture LC-MS and TR-IFMA methods^45^. Good accordance among ProGRP values is demonstrated. These results demonstrate very clearly the suitability of the new MISPE-LC-MS/MS method for the extraction and quantification of ProGRP present in clinical serum samples.

## Conclusion

In this paper, a template analogue imprinting strategy was implemented successfully for the design and synthesis of a polymeric synthetic receptor enabling biomarker determination in native serum at the pM level. Precipitation polymerization was used to deliver molecularly imprinted polymer microspheres in a physical format very convenient for their direct packing into trap column and direct integration with an LC-MS system for automated extraction of the ProGRP signature peptide. A MIP synthesized using EAMA.HCl as the sole functional monomer was found to be especially promising for the retention of the target peptide, and so was evaluated in further detail.

Coupling of a MIP trap column with an analytical column and tandem MS detection allowed for the development of the first automated method for the determination of ProGRP in patient samples. The practical combination of a low sample volume (50 μL) and short analysis time represents a noteworthy breakthrough in ProGRP determination by LC-MS using synthetic receptors. In addition, the low limits of detection and quantification were achieved without the need for antibodies and this is a unique novelty in ProGRP analysis.

Future studies should focus on a rapid and automated protein digestion before the MISPE in order to increase further the clinical advantage of the platform presented in this paper.

## Methods

### Reagents

The peptide template Z-NLLGLIEA[Nle] (purity 96.58%), was purchased from LifeTein, *N*-(2-aminoethyl)methacrylamide hydrochloride (EAMA.HCl, purity ≥98%) was purchased from Polysciences Inc. (Niles, IL, USA), *N*-3,5-*bis*(trifluoromethyl)-phenyl-*N*′-4-vinylphenylurea (purity >95%) is not commercially available and was kindly donated by Dortmund University, 2,2′-Azo*bis*isobutyronitrile (AIBN, purity ≥98%) was purchased from BDH (UAE). Divinylbenzene-80 (DVB-80, 80% DVB isomers and 20% ethylvinylbenzene isomers), 1,2,2,6,6-pentamethylpiperidine (PMP, purity >99%), tetrabutylammonium hydroxide solution (TBA.HO, 1.0 M in methanol, 25%≤ purity <50%) and hydrochloric acid (purity ≥37%) were all purchased from Sigma-Aldrich (St. Louis, MI, USA). DVB-80 was purified by filtration through a short plug of neutral aluminium oxide prior to use. AIBN was recrystallized from acetone at low temperature. All other chemicals used (acetonitrile (MeCN), methanol and dimethyl sulfoxide [DMSO, purity ≥99.9%]) were of analytical grade.

### Protein and Peptide Standards

Recombinant ProGRP isoform 1 (AA 1−125 + 8) was cloned from human cDNA (OriGene Technologies, Rockville, MD,USA), expressed in Escherichia coli (Promega, Madison, WI, USA) using pGEX-6P-3 constructs (GE Healthcare Little Chalfont, UK) and purified as described elsewhere^46^. Solutions of ProGRP and the Internal Standard (IS) NLLGLIEA[K_^13^C_6_^15^N_2_] (purity >95%, Sigma-Aldrich) were prepared as described elsewhere^21^.

### Serum Samples

Human serum from healthy subjects was obtained from Ullevål Hospital (Oslo, Norway), and serum samples from cancer patients were supplied by Radiumhospitalet (Oslo, Norway). All serum samples were stored at −30 °C. The use of patient samples for research purposes was approved by the Norwegian Regional Committee for Medical Research Ethics (REK, http://helseforskning.etikkom.no). Informed consent was obtained from all subjects. Methods used to analyse all serum samples were in accordance with relevant guidelines and regulations.

### Synthesis, Characterization and Column Packing of MIPs and NIPs

Four distinct polymers were synthesized after protocol optimization (Supplementary section 1): two MIPs (MIP A and B) and two corresponding non-imprinted polymers (NIPs) (NIP A and B). MIPs were synthesized by firstly adding the template Z-NLLGLIEA[Nle] (8.2 mg, 7 μmol) into a borosilicate Kimax tube. Thereafter, DMSO (1 mL) was added (to dissolve the template), followed by PMP (1.9 mg, 0.01 mmol) and the functional monomer EAMA.HCl (12.3 mg, 0.07 mmol). For the synthesis of MIP B, TBA.HO (3.93 mg, 0.01 mmol) and *N*-3,5-*bis*(trifluoromethyl)-phenyl-N′-4-vinylphenylurea (5.25 mg, 0.01 mmol) were also included. MeCN (24 mL) was then added followed by DVB-80 (0.49 g, 0.53 mL, 3.73 mmol) and AIBN (22.3 mg, 0.2 mmol). (For the synthesis of the NIPs, the template was omitted from the synthetic protocols). The four solutions were then ultrasonicated for 10 minutes at ambient temperature and purged with oxygen-free nitrogen gas for 10 minutes at ice-bath temperature, to remove dissolved molecular oxygen. Thereafter, the reaction vessels were sealed under nitrogen and transferred to a Stuart Scientific S160 incubator equipped with a Stovall low-profile roller. The incubator temperature was ramped from ambient to 60 °C over a period of around two hours and then maintained at 60 °C for a further 46 hours to yield milky suspensions of polymer microspheres. Finally, the polymer microspheres were isolated from the reaction media by filtration on 0.45 μm nylon membrane filters, and washed sequentially with MeCN (50 mL), MeOH/0.1 M aq. HCl (90/10, v/v, 50 mL) and MeOH (50 mL), and finally dried overnight in Townson & Mercer vacuum oven at 70 °C.

The microspheres were evaluated in terms of their size and size distribution. Scanning Electron Microscopy (SEM) images were acquired using a Stereoscan 90 (Cambridge Instruments). Polymer microspheres were sputter-coated with gold using a Polar SC500A Sputter Coater Fison Instrument prior to analysis. Image analysis of the SEM micrographs was performed using Image J^47^ software, on a population of 100 microspheres.

Brunauer-Emmett-Teller (BET) surface area analysis and Barrett-Joyner-Halenda (BJH) pore size and volume analysis were assessed by using an ASAP 2000 BET Analyzer. For each analysis, around 0.2 g of polymer was dried overnight in a vacuum oven at 70 °C, followed by a degassing step (pressure ~3 mmHg) for 24 h at 100 °C. BET theory was applied for the determination of specific surface areas, BJH cumulative adsorption pore volume was determined for pores between 1.7 and 300 nm, the micropore volume was based on the Harkins and Jura’s thickness equation^48^.

Particles were evaluated in terms of binding capacity by plotting of the binding isotherms and the calculation of imprinting factors (Supplementary section 3.2 and 3.3, respectively). For both MIP/NIP pairs, binding isotherms were fitted by Freundlich curves as decribed by Rampey *et al*.^49^ and imprinting factors of the polymers were calculated as described by Manesiotis *et al*.^50^. Packing of the MIPs and NIPs in trap columns (1.4 × 5mm with 1 μm stainless steel frits) was performed by G&T Septech, Norway, by wet packing around 10 mg of polymer in 1200 μL of MeCN using a flowrate of 500 μL/min. In order to verify the quality of the packing of the columns, transversal microscopy (Dino Capture microscope with × 100 magnification) images were acquired, and backpressures measured when flowing a mobile phase of 70% MeCN in water at 50 μL/min.

### On-Line MISPE-Liquid Chromatography-Tandem Mass Spectrometry (MISPE-LC-MS/MS) analysis

The LC system consisted of an LPG-3400 M binary pump with degasser, an ISO-3100 A loading pump, a WPS-3000TRS autosampler and FLM-3000 flow-manager (all Dionex, Sunnyvale, CA, USA). The LC system was controlled by Chromeleon v. 6.80 SR6 (Dionex). The extraction was carried out by using the MIP A trap column. The LC separation was carried out using a Hypersil GOLD aQ, analytical column (Thermo Scientific, 100 Å, 3 μm, 1 × 50 mm) preceded by a Hypersil GOLD aQ Drop-In Guard Cartridge (Thermo Scientific, 100 Å, 3 μm, 1 × 10 mm).

The extraction was performed by loading 25 μL of sample with the loading buffer (20 mM aqueous formic acid [FA]). The isocratic flow (30 μL/min) was directed to the waste *via* the MIP cartridge, as shown in Fig. 5A. After 10 minutes, the system was switched in order to forward-flush the MIP cartridge to the analytical column and thus to the MS detector, as shown in Fig. 5B. The gradient flow (30 μL/min) had an initial ratio of mobile phase A (20 mM FA) to mobile phase B (pure MeCN) of 95:5 (v/v); this was kept constant for 10 minutes before the elution using a 27 minute linear gradient from 5 to 86% of mobile phase B. After the gradient run, the MIP column and the analytical column were washed for 5 minutes with 97% mobile phase B and re-equilibrated with mobile phase A, as shown in Fig. 5C.

The MS system consisted of a TSQ Quantum Access (Thermo Scientific) and was used for quantification of signature peptides by Selected Reaction Monitoring (SRM) experiments. The following transition pairs were monitored (qualifier and quantifier, respectively): for the ProGRP signature peptide NLLGLIEAK (485.8 → 630.3 and 485.8 → 743.4); for its internal standard NLLGLIEA[K_^13^C_6_^15^N_2_] (489.9 → 638.3 and 489.9 → 751.4); for the ProGRP isoform 1 signature peptide LSAPGSQR (408.2 → 272.6 and 408.2 → 544.4).

TSQ-data were processed by Xcalibur’sTM QualBrowser (Thermo Scientific) and peak areas, automatically processed by the Genesis peak detection algorithm, were used for the evaluation of the MS-responses. Only peaks with signal-to-noise (S/N) ratios above 10 and retention times and ion ratios corresponding to that of standard samples were considered.

### ProGRP digestion

ProGRP isoform 1 was diluted to the desired concentration with 50 mM freshly prepared ammonium bicarbonate buffer (ABC), trypsin added at an enzyme-to-substrate ratio of 1:40 (w/w), and then incubated at 37 °C overnight at 800 r.p.m.

### Calibration curve and patient sample analysis

For the calibration curve, triplicates of human serum (50 μL) were spiked with ProGRP isoform 1 and vortexed for 30 seconds, to give the desired final concentrations: 0.183, 1.83, 3.66, 7.32, 36.6, 73.2 and 110 nM. Protein precipitation was performed by adding a volume of cold MeCN (−32 °C) to the serum (MeCN to serum v/v ratio = 0.75:1) and shaking for 5 minutes. Samples were then centrifuged at 10,000 r.p.m. for 10 minutes and the supernatants evaporated to dryness under a nitrogen stream at 40 °C. 50 μL of the trypsin solution (1 mg/mL in 50 mM ABC buffer) (1:40 protein: enzyme ratio) was used to reconstitute the samples and tryptic digestion was performed at 37 °C overnight. Analysis of patient samples (Oslo University Hospital, Oslo, Norway; approved by the Norwegian Regional Committee for Medical Research Ethics REK, http://helseforskning.etikkom.no) was performed by preparing the samples in triplicate as described for the calibration curve without the spiking of ProGRP isoform 1. All the samples were spiked with IS 10 nM before the injection to the chromatographic system.

## Additional Information

**How to cite this article:** Rossetti, C. *et al*. Automated Protein Biomarker Analysis: on-line extraction of clinical samples by Molecularly Imprinted Polymers. *Sci. Rep.* **7**, 44298; doi: 10.1038/srep44298 (2017).

**Publisher's note:** Springer Nature remains neutral with regard to jurisdictional claims in published maps and institutional affiliations.

## Supplementary Material

Supplementary Information

## Figures and Tables

**Table 1:** Benchmarking of ProGRP concentrations in patient samples measured by the three analytical methods.

	MISPE-LC-MS	immuno-LC-MS^45^	TR-IFMA^45^
Patient_39	2402 pM	922 pM	2425 pM
Patient_43	1029 pM	918 pM	1899 pM
