# Supplementary material for: Automated Protein Biomarker Analysis: on-line extraction of clinical samples by Molecularly Imprinted Polymers
Source: Sci Rep. 2017 Mar 17;7:44298. doi: 10.1038/srep44298 (PMC5355873; doi:10.1038/srep44298)
Supplement: Supplementary Information [file srep44298-s1.pdf]

SUPPORTING INFORMATION

Automated Protein Biomarker Analysis: on-line extraction of clinical samples by Molecularly Imprinted Polymers

Cecilia Rossetti[1], Magdalena A. Świtnicka-Plak[2], Trine Grønhaug Halvorsen[1], Peter A.G. Cormack[2], Börje Sellergren[3] and Léon Reubsaet*[1]

1. Department of Pharmaceutical Chemistry, University of Oslo, School of Pharmacy, Postbox 1068 Blindern, 0316 Oslo, Norway

2. WestCHEM, Department of Pure and Applied Chemistry, University of Strathclyde, Thomas Graham Building, 295 Cathedral Street, Glasgow, G1 1XL, United Kingdom

3. Department of Biomedical Sciences, Faculty of Health and Society, Malmö University, SE20506 Malmö, Sweden.

TABLE OF CONTENTS

1. **Optimization of the polymer synthesis:**

1.1 Solubility tests.

Table S-1 Solubility tests for EAMA.HCl S2

1.2 Choice of reaction vessel and synthesis conditions.

Table S-2 Synthesis conditions and the yields of the non-imprinted polymers S2

Figure S-1: Overlap of the FTIR spectra of NIP 1 and NIP 4 S3

2. **Synthesis of the polymers:**

Table S-3 Monomer feed conditions and yields of the imprinted polymers S3

3. **Characterization of the polymers:**

3.1 SEM analysis.

Figure S-2: SEM images of polymers S4

3.2 BET analysis.

Table S-4 Nitrogen sorption data S4

3.3 Binding isotherms.

Figure S-3 Equilibrium binding isotherms for MIP/NIP A S5

Figure S-4 Equilibrium binding isotherms for MIP/NIP B S5

Table S-5: Freundlich fitting parameters for all the polymers S5

3.4 Imprinting factors.

Table S-6 Retention coefficients and imprinting factors S6

4. **Method development:**

4.1 Drop of peptide signal intensity due to increased pH.

Figure S-5: Direct MS infusion TIC chromatogram of 1 nM NLLGLIEA[K_$^{13}C_6$ $^{15}N_2$] S6

4.2 Linearity of the method.

Figure S-6: Calibration curve S7

5. **Verification of NIP failure:**

Figure S-5: Extraction on NIP A of a serum samples spiked with ProGRP iso1 S7

REFERENCES S7

1. Optimization of the polymer synthesis:

1.1 Solubility tests.

In order to adapt the experimental approaches described elsewhere[1] to the precipitation polymerization procedure, solubility tests for the functional monomer EAMA.HCl were performed. Moreover, the solubility tests had the aim to determine the amount of DMSO needed to bring all the precipitation polymerization components into a homogenous solution. Different mole ratio of EAMA.HCl and DVB-80 (crosslinker) together with different combinations of MeCN (solvent) and DMSO (co-solvent) were tested, as presented in Table S-1.

Table S-1 Solubility tests for EAMA.HCl to be co-polymerized with DVB in MeCN and DMSO.

Test #	EAMA.HCl / DVB (mol ratio)	EAMA.HCl (mmol)	MeCN / DMSO (v/v)
**1**	1 / 5	0.7470	99 / 1
**2**	1 / 5	0.7470	96 / 4
**3**	0.1 / 5	0.0747	99 / 1
**4**	0.1 / 5	0.0747	96 / 4

The use of EAMA.HCl in the same 1 / 5 molar ratio of EAMA.HCl / DVB as used in the synthesis performed in the previous work[1] resulted in monomer insolubility. This problem was overcome by decreasing the functional monomer concentrations. In solubility test #4, the amount of EAMA.HCl was reduced ten times and was dissolved completely in 4 % of DMSO and 96 % of MeCN. Thus the mole ratio of EAMA.HCl to DVB was set at 0.1:5. The mole ratio of DVB to *N*-3,5-*bis*(trifluoromethyl)-phenyl-N'-4-vinylphenylurea was set at 5:0.02.

1.2 Choice of reaction vessel and synthesis conditions.

In order to optimize the synthetic protocol, polymers without the addition of the template (non-imprinted polymers) were pre-tested as shown in Table S-2. A polymer with only the crosslinker (DVB-80) was prepared as control and polymers providing the use of the selected functional monomers (EAMA.HCl and *N*-3,5-*bis*(trifluoromethyl)-phenyl-*N*'-4-vinylphenylurea) were prepared in two different reaction vessels.

Table S-2 Synthesis conditions and the yields of the non-imprinted polymers prepared in different reaction vessel.

Polymer Code	Reaction vessel	Functional Monomer (mmol)	DVB-80 (mmol)	Solvent (mL)	AIBN (mol%)	Incubation time	Other components (mmol)	Polymer Yield (%)
**NIP 1**	Borosilicate Kimax tube	-	3.06	MeCN (20)	2	24 h	-	29
**NIP 2**	Borosilicate Kimax tube	EAMA.HCl (0.07)	3.73	MeCN (24) DMSO (1)	2	24 h	PMP (0.006)	22
**NIP 3**	Polyethylene Nalgene bottle	EAMA.HCl (0.07)	3.73	MeCN (24) DMSO (1)	2	24 h	PMP (0.006)	19
**NIP 4**	Borosilicate Kimax tube	EAMA.HCl (0.07) urea monomer* (0.01)	3.73	MeCN (24) DMSO (1)	2	24 h	PMP (0.006) $TBA.HO^-$(0.007)	28
**NIP 5**	Polyethylene Nalgene bottle	EAMA.HCl (0.07) urea monomer* (0.01)	3.73	MeCN (24) DMSO (1)	2	24 h	PMP (0.006) $TBA.HO^-$(0.007)	4

* *N*-3,5-*bis*(trifluoromethyl)-phenyl-*N*'-4-vinylphenylurea.

As the table shows, yields are lower for the polymers synthesized in Polyethylene Nalgene bottles. Thus Borosilicate Kimax tubes were used to perform the syntheses. PMP and TBA.HO were used to bring the various functional groups (of EAMA and *N*-3,5-*bis*(trifluoromethyl)-phenyl-N'-4-vinylphenylurea respectively) into appropriate ionization states for non-covalent interactions between functional monomers and the template (which here was not added). Moreover, for the synthesis of Molecularly Imprinted Polymers, where the template will be added at the beginning, it was decided to increase the amount of PMP and $TBA.HO^-$ from 0.006 mmol to 0.01 mmol since the template has two sites able to bind the functional monomers: the carboxylic acid group in the glutamic acid (E) residue and C-terminus of Z-NLLGLIEA[Nle] as shown in Figure 2 of the main text.
The incubation time was extended to 48 hours in order to increase the yield of the polymerization as Table S-3 demonstrates.
A control polymer made of DVB-80 only gave a reaction yield typical for the polymerization of divinylbenzene under such precipitation polymerization conditions.
For this polymer (NIP 1) as for the polymers NIP 2 and 4, the FTIR spectra were acquired using a Shimadzu IRAffinity-1 Fourier Transform Infrared Spectrophotometer with Attenuated Total Reflection (ATR) Mode.

Figure S-1 shows the Overlap of the FTIR spectra of NIP 1 [poly(EVB-co-DVB-80)] (black solid line) and NIP 4. [poly(*N*-3,5-*bis*(trifluoromethyl)-phenyl-N'-4-vinylphenylurea-co-EAMA.HCl-co-DVB-80)] (orange solid line) and presents the characteristic peaks for the polymerization of DVB-80: aromatic C-H stretches at 3018 cm^{-1} and 3007 cm^{-1}, aliphatic C-H stretches at 2916 cm^{-1}, aromatic C=C stretches at 1627 cm^{-1}, 1600 cm^{-1} and 1510 cm^{-1}, alkene stretches at 987 cm^{-1} and 902 cm^{-1}, and three stretches at 829 cm^{-1}, 794 cm^{-1} and 709 cm^{-1}, corresponding to para- and meta-di-substituted benzene rings.

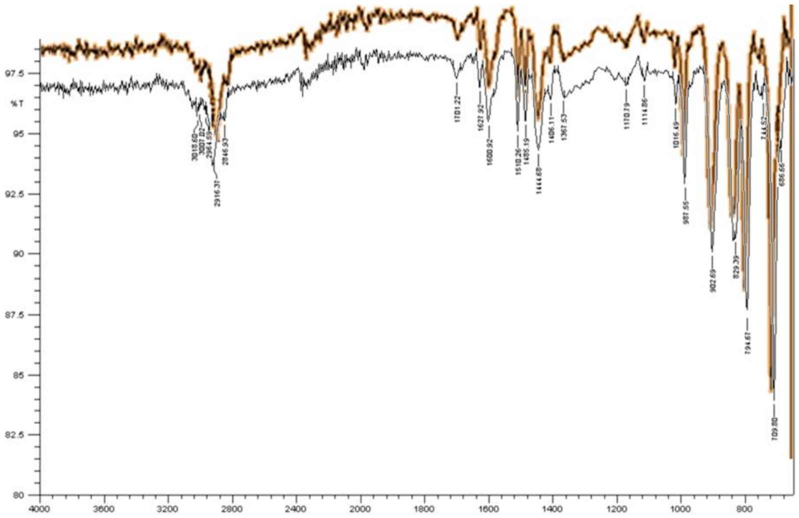

Figure S-1: Overlap of the FTIR spectra of NIP 1 [poly(EVB-co-DVB-80)] (black solid line) and NIP 4. [poly(N-3,5-bis(tTrifluoromethyl)-phenyl-N'-4-vinylphenylurea-co-EAMA.HCl-co-DVB-80)] (orange solid line).

Both poly(EVB-co-DVB-80) and NIP 4. [poly(*N*-3,5-*bis*(trifluoromethyl)-phenyl-N'-4-vinylphenylurea-co-EAMA.HCl-co-DVB-80)] showed these peaks only. Also, NIP 2 [poly(EAMA.HCl-co-EVB-co-DVB-80)] showed the typical peaks associated with the poly(EVB-co-DVB-80) only. Peaks associated with the presence of EAMA.HCl or *N*-3,5-*bis*(trifluoromethyl)-phenyl-N'-4-vinylphenylurea were not observed, as the amount of functional monomers present in the monomer feed was relatively small compared to the level of DVB.

For this reason, elemental microanalysis of the polymers was not performed, since the elemental composition was expected to reflect the poly(EVB-co-DVB-80) composition. However, the theoretical composition of polymers produced could be determined from the composition and the reactivity of the monomers used in the polymerizations performed.

2. Synthesis of the polymers:

Table S-3 Monomer feed conditions and the yields of the polymeric products: Z-NLLGLIEA[Nle] imprinted polymers and their corresponding NIPs.

Polymer Code	Template[a] (mmol)	Functional Monomer (mmol)	DVB-80 (mmol)	Solvent (mL)	AIBN (mol%)[#]	PMP or TBA.HO[a] (mmol)	Incubation time (h)	Polymer Yield (%)
**NIP A**	-	EAMA.HCl (0.07)	3.73	MeCN (24) DMSO (1)	2	PMP (0.01)	48	54
**MIP A**	0.007	EAMA.HCl (0.07)	3.73	MeCN (24) DMSO (1)	2	PMP (0.01)	48	41
**NIP B**	-	EAMA.HCl (0.07) urea monomer* (0.01)	3.73	MeCN (24) DMSO (1)	2	PMP (0.01) TBA.HO (0.01)	48	49
**MIP B**	0.007	EAMA.HCl (0.07) urea monomer* (0.01)	3.73	MeCN (24) DMSO (1)	2	PMP (0.01) TBA.HO (0.01)	48	52

* *N*-3,5-*bis*(trifluoromethyl)-phenyl-*N*'-4-vinylphenylurea. [#] Relative to polymerizable double bonds.

[a] PMP and TBA.HO were used to bring the various functional groups into appropriate ionization states for non-covalent interaction and to promote template solubility.

3. Characterization of the polymers:

3.1 SEM analysis.

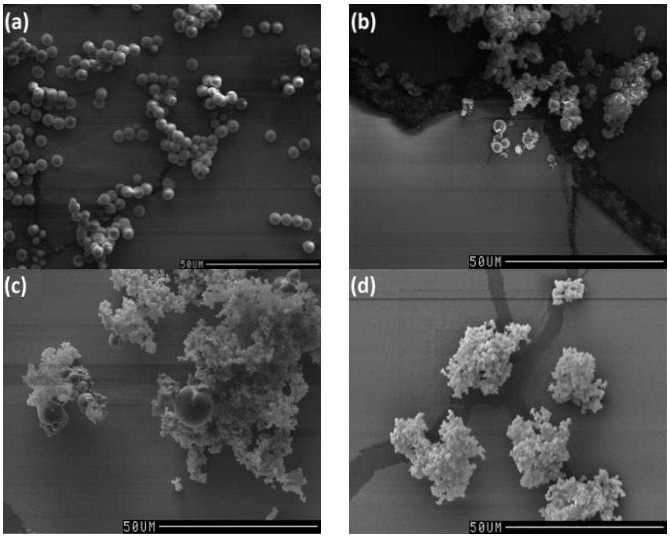

Figure S-2: SEM images of the polymers: (a) NIP A, (b) MIP A, (c) NIP B, (d) MIP B (x 777 magnification).

3.2 BET analysis.

Table S-4 Nitrogen sorption data for the non-imprinted and molecularly imprinted polymers for the Z-NLLGLIEA[Nle] target.

Polymer Code	BET specific surface area (m^2/g)	Specific pore volume (cm^3/g)	Average pore diameter[a] (nm)	Micropore volume (cm^3/g)	Micropore area (m^2/g)
**NIP A**	221	0.036	2.10	0.060	137
**MIP A**	31	n/a	n/a	0.032	76
**NIP B**	307	0.026	1.96	0.099	218
**MIP B**	349	0.083	2.34	0.093	205

[a]The average pore diameter was determined by equation : Average pore diameter= $\frac{\text{4 pore volume}}{\text{BET surface area}}$

3.3 Binding isotherms.

The polymers (1 mg) were mixed with different solution of NLLGLIEA[K_$^{13}C_6{}^{15}N_2$] spanning the concentration range of 10 – 220 nM in 700 µL of MeCN: ABC buffer (5:95) (50 mM pH 7.6), and were incubated for 24 h at 20 °C setting the Eppendorf shaker at 800 r.p.m. Afterward the solutions were centrifuged at 10000 r.p.m. for 30 minutes and the supernatants were collected and injected into the LC-MS/MS system.

The chromatographic separation was carried out by using Hypersil GOLD aQ, analytical column (Thermo Scientific, 100 Å, 3 µm, 1 × 50 mm) preceded by a pre-column (Hypersil GOLD aQ Drop-In Guard Cartridge Thermo Scientific, 100 Å, 3µm, 1 × 10 mm). The 30 min linear gradient ranged from 1 to 85% of mobile phase B (20 mM FA : MeCN 5:95, v/v) and the column was re-equilibrated with 99% of mobile phase A (20 mM FA : MeCN 95:5, v/v). The column temperature was kept constant at 30 °C. A triple quadrupole (TSQ Quantum™ Access, Thermo Fisher Scientific) was used for quantification of the peptide in Selected Reaction Monitoring (SRM) mode, following a transition pair: 489.9 → 638.3 and 489.9 → 751.4. Peak areas, automatically processed by genesis peak detection algorithm, were calculated by Xcalibur'sTM QualBrowser (Thermo Scientific).

The amount of peptide bound to the polymer (B) was calculated by subtracting the non-bound analyte (F) from the initial NLLGLIEA[K_13$C_6$15N_2] concentration in the solution. The incubation was performed in duplicate. The curves were obtained by plotting the average of B versus F (Figure S-2 and S-3), and then fitted to the Freundlich model using the following power function:

$$B = aF^m \qquad \text{Eq. 1}$$

with *B* the concentration of bound analyte and *F* the amount of free analyte. *a* and *m* are parameters which describe the power function and were used to calculate the average affinity constant *K* as described from Rampey et al[2].

$$K = \left(\frac{m}{m-1}\right)\frac{K_1^{1-m}-K_2^{1-m}}{K_1^{-m}-K_2^{-m}} \qquad \text{Eq. 2}$$

with $K_1 = 1/F_{max}$ and $K_2 = 1/F_{min}$;

$$N = \mathrm{a}\,(1 - \mathrm{m}^2)(\mathrm{K}_1^{-m} - \mathrm{K}_2^{-m}) \qquad \text{Eq. 3}$$

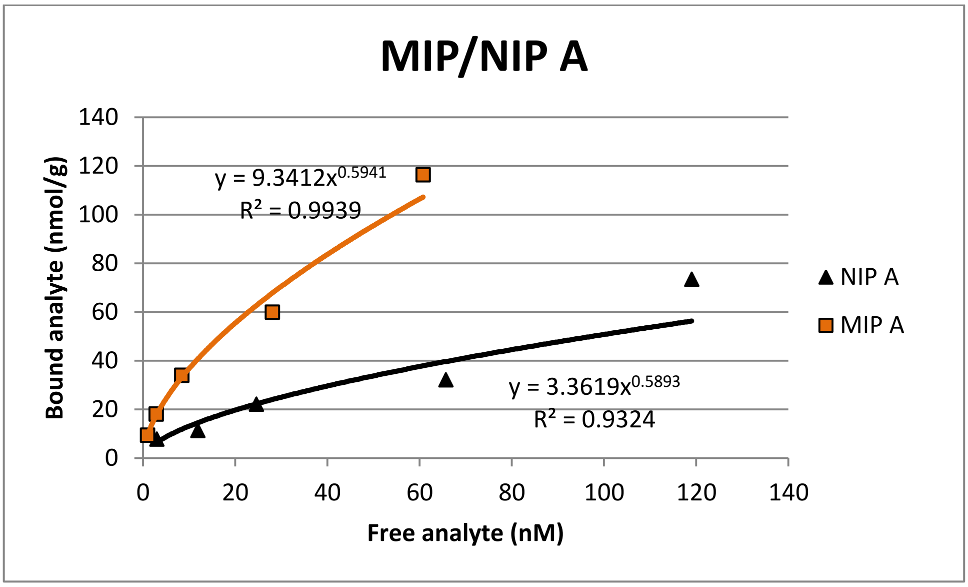

Figure S-3: Equilibrium binding isotherms obtained from Freundlich fitting for the uptake of NLLGLIEA[K_$^{13}C_6{}^{15}N_2$] by MIP A (squares, orange line) and NIP A (triangles, black line) in ABC/MeCN (95:5, v/v).

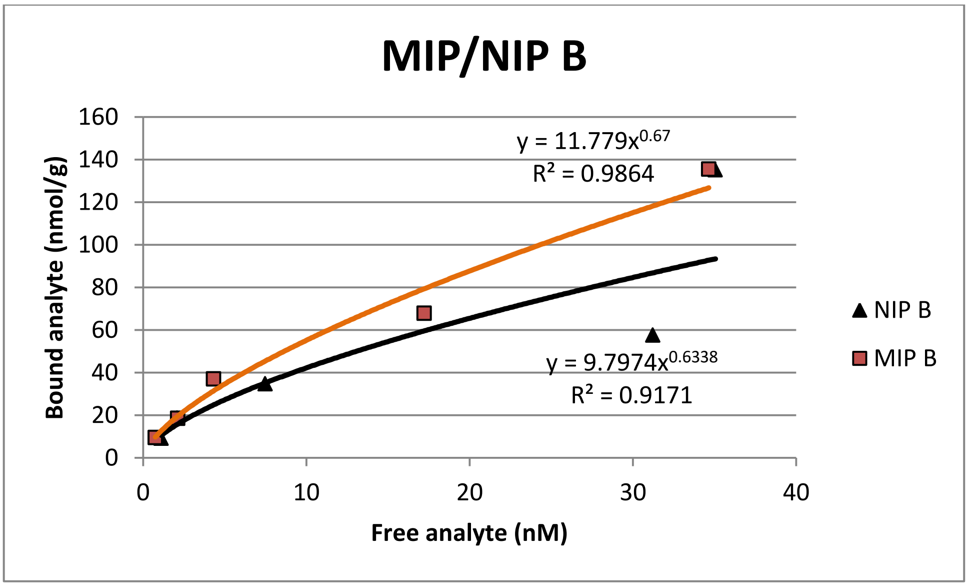

Figure S-4: Equilibrium binding isotherms obtained from Freundlich fitting for the uptake of NLLGLIEA[K_$^{13}C_6{}^{15}N_2$] by MIP B (squares, orange line) and NIP B (triangles, black line) in ABC/MeCN (95:5, v/v).

Table S-5: Freundlich fitting parameters for all the polymers

	**Affinity constant, K** (nM^{-1})	**Total Number of binding sites, N** ($\mu mol\ g^{-1}$)	**Heterogeneity parameter , m**[a]	**Binding capacity, a**	**Regression coefficient, R^2**
**MIP A**	0.11	6.73	0.59	9.34	0.99
**NIP A**	0.05	9.00	0.59	3.36	0.93
**MIP B**	0.16	5.40	0.67	11.78	0.99
**NIP B**	0.14	4.64	0.63	9.80	0.92

a The parameter *m* represents the heterogeneity index of the polymer, ranging from 1 (homogeneous samples) to 0 (heterogeneous samples).

The Freundlich model commonly describes site distributions well in MIPs. The model implies a heterogenous distribution of sites continuously ranging from low to high binding energies and absence of homogenous populations of binding sites The parameter m is of particular importance and here confirms an heterogeneous population of molecularly imprinted binding sites arising from the non-covalent molecular imprinting strategy adopted.

3.3 Imprinting Factors.

Imprinting factors of the polymers were calculated as described by Manesiotis *et al.*[3] for both MIP/NIP pairs based on the retention times of a non-retained peptide (LSAPGSQR) and the target analyte (NLLGLIEAK) after the isocratic elution with 5% MeCN from the cartridges according the equation 4:

$$\mathrm{IF} = \mathrm{k'_{MIP}}/\mathrm{k'_{NIP}} \qquad \text{Eq.4}$$

where $\mathrm{k'_{MIP}}$ and $\mathrm{k'_{NIP}}$ are the respective retention factors defined as:

$$\mathrm{k'} = (\mathrm{t_R} - \mathrm{t_0})/\mathrm{t_0} \qquad \text{Eq.5}$$

with $\mathrm{t_R}$ the retention time of the analyte (NLLGLIEAK) and $\mathrm{t_0}$ the retention time of a not-retained peptide (LSAPGSQR).

Table S-6: Retention coefficients and imprinting factors of the two polymerisation protocols.

	Retention factor, k'	Imprinting factor, IF
**MIP A**	14.36	1.11
**NIP A**	12.91	
**MIP B**	13.41	1.00
**NIP B**	13.43	

4. Method development:

4.1 Drop of peptide signal intensity due to increased pH.

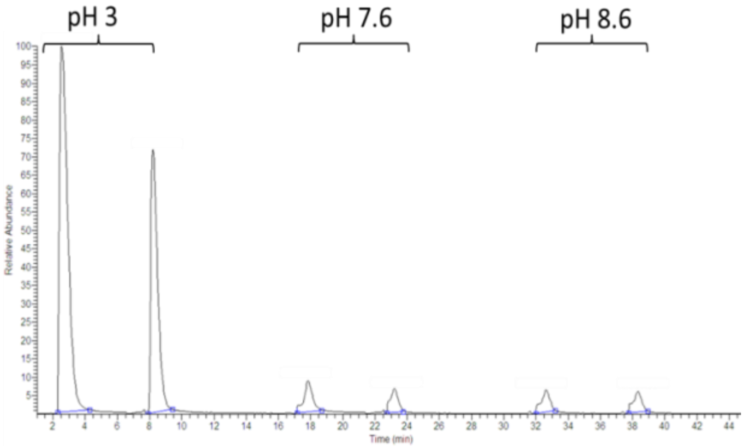

Figure S-5: Direct MS infusion TIC chromatogram of 1 nM NLLGLIEA[K_$^{13}C_6{}^{15}N_2$] prepared at different pH values and injected at different time points

Figure S-5 shows the peaks obtained when the different peptide solutions were injected in duplicate (no MIP or analytical columns): The highest intensity was registered at pH 3.0, whilst the drop in signal intensity is significant at pH ≥ 7.6.

4.2 Linearity of the method.

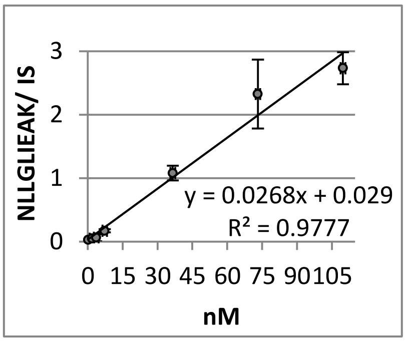

Figure S-6: Calibration curve obtained plotting the ratio of the area of the signature peptide NLLGLIEAK to the area of the internal standard (IS) for different ProGRP isoform 1 concentrations in serum.

5. Verification of NIP failure:

The extraction on a NIP cartridge (NIP A) of a serum samples spiked with 1 nM of ProGRP isoform 1 was performed in order to compare the performance with the extracted samples from the calibration curve. Addition of a solution of NLLGLIEA[K_$^{13}C_6{}^{15}N_2$] 10 nM was performed before the injection in the chromatographic system in order allow a correct peak identification.

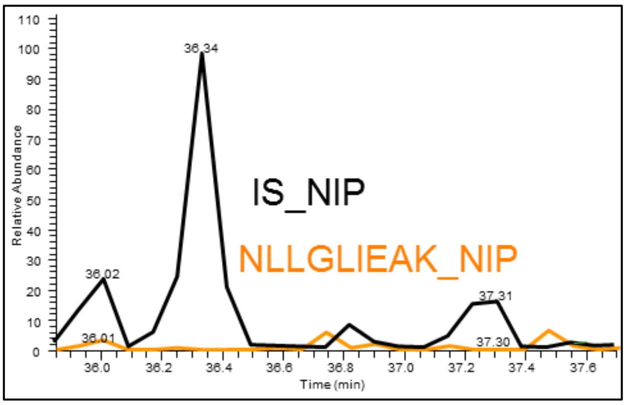

Figure S-7: Extraction on NIP A of a serum samples spiked with of ProGRP iso1 (1 nM) (orange line). Addition of a solution of NLLGLIEA[K_$^{13}C_6{}^{15}N_2$] 10 nM (black line).

The presence of the internal standard only show the impossibility of the NIP in enriching the targeted peptide within the serum sample after the optimized sample preparation, while the MIP could enrich such concentration with similar intensities for both target peptide and internal standard.

REFERENCES

1 Qader, A. A. *et al.* Peptide imprinted receptors for the determination of the small cell lung cancer associated biomarker progastrin releasing peptide. *Journal of Chromatography A* **1370**, 56-62, doi:10.1016/j.chroma.2014.10.023 (2014).

2 Rampey, A. M. *et al.* Characterization of the Imprint Effect and the Influence of Imprinting Conditions on Affinity, Capacity, and Heterogeneity in Molecularly Imprinted Polymers Using the Freundlich Isotherm-Affinity Distribution Analysis. *Analytical Chemistry* **76**, 1123-1133 (2004).

3 Manesiotis, P., Kashani, S. & McLoughlin, P. Molecularly imprinted polymers for the extraction of imiquimod from biological samples using a template analogue strategy. *Analytical Methods* **5**, 3122-3128, doi:10.1039/c3ay40239h (2013).
